# Supplementary figures and images for: In silico epitope prediction and evolutionary analysis reveals capsid mutation patterns for enterovirus B
Source: PLoS One. 2023 Aug 28;18(8):e0290584. doi: 10.1371/journal.pone.0290584 (PMC10461833; doi:10.1371/journal.pone.0290584)

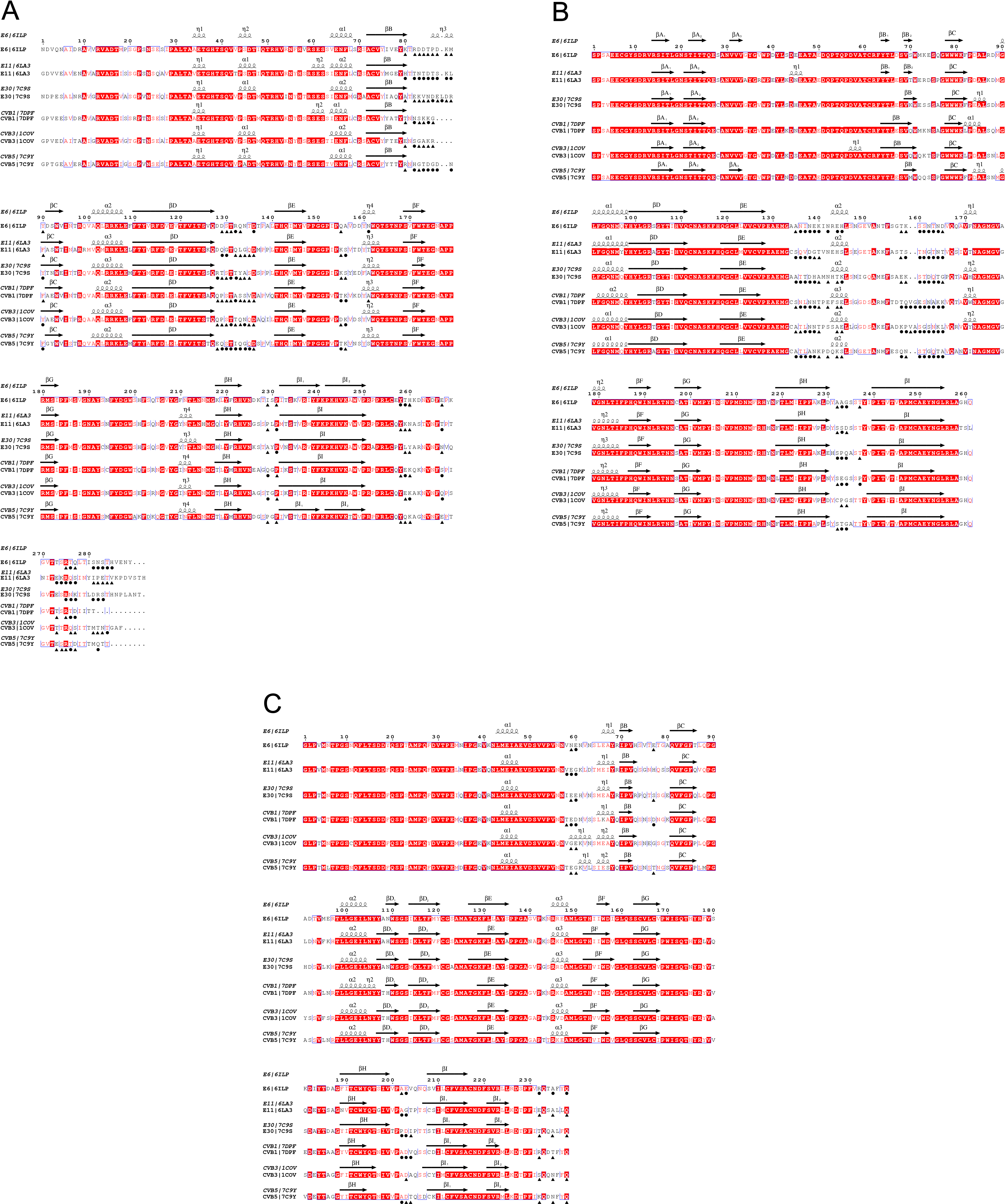

Supplement: S1 Fig — Sequence alignments of VP1 (A), VP2 (B) and VP3 (C). The secondary structure elements (β-sheets and α-helices) are shown above the corresponding alignments as arrows (β-sheets) and spirals (α-helices), respectively. Predicted epitopes are shown as black circles (core epitopes) and black triangles (surrounding epitopes). (TIF) [file pone.0290584.s001.tif]

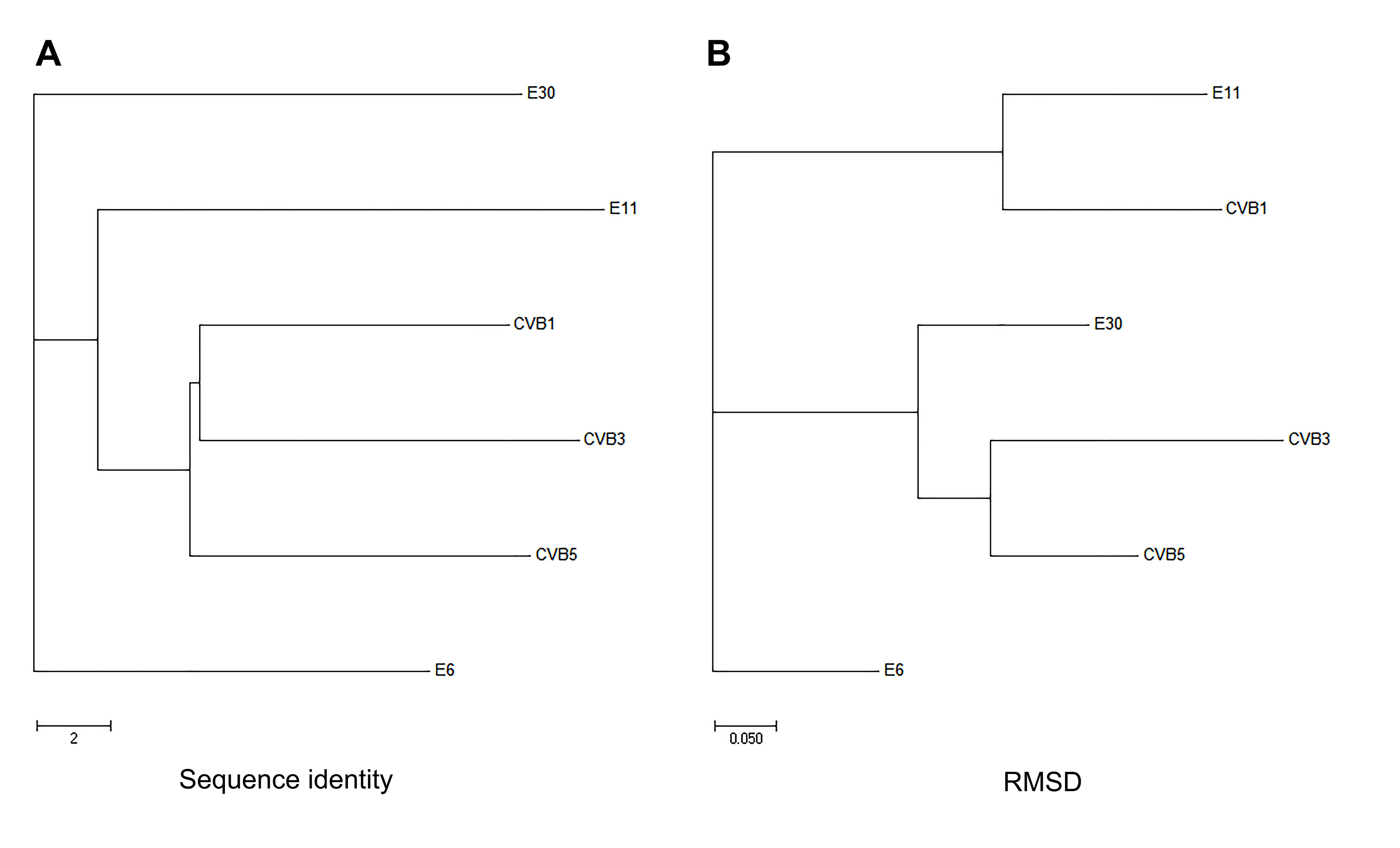

Supplement: S2 Fig — (TIF) [file pone.0290584.s002.tif]

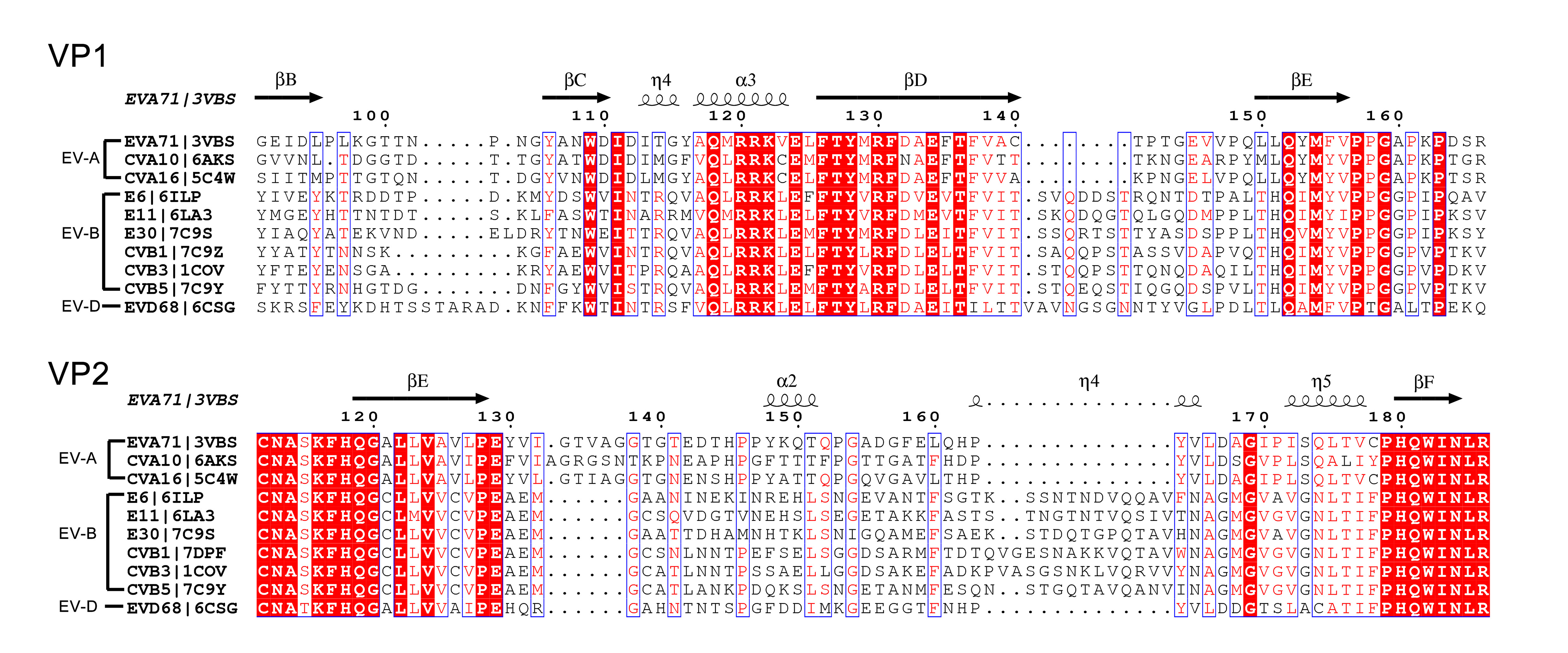

Supplement: S3 Fig — The secondary structure is labeled the same as in S1 Fig. (TIF) [file pone.0290584.s003.tif]

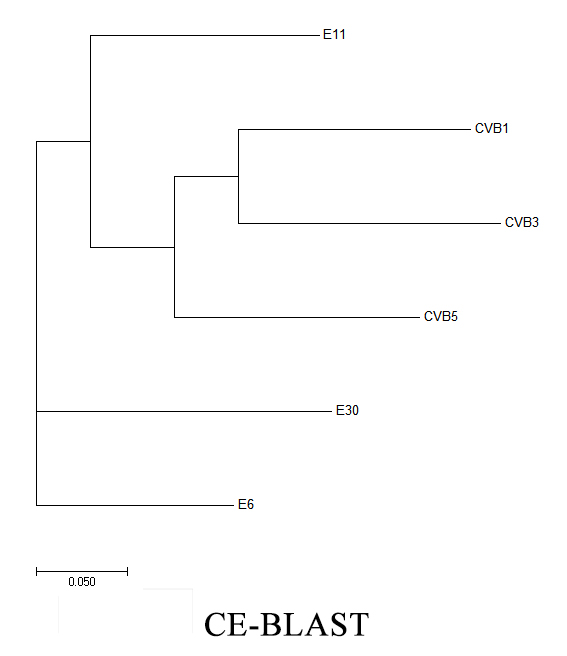

Supplement: S4 Fig — (TIF) [file pone.0290584.s004.tif]

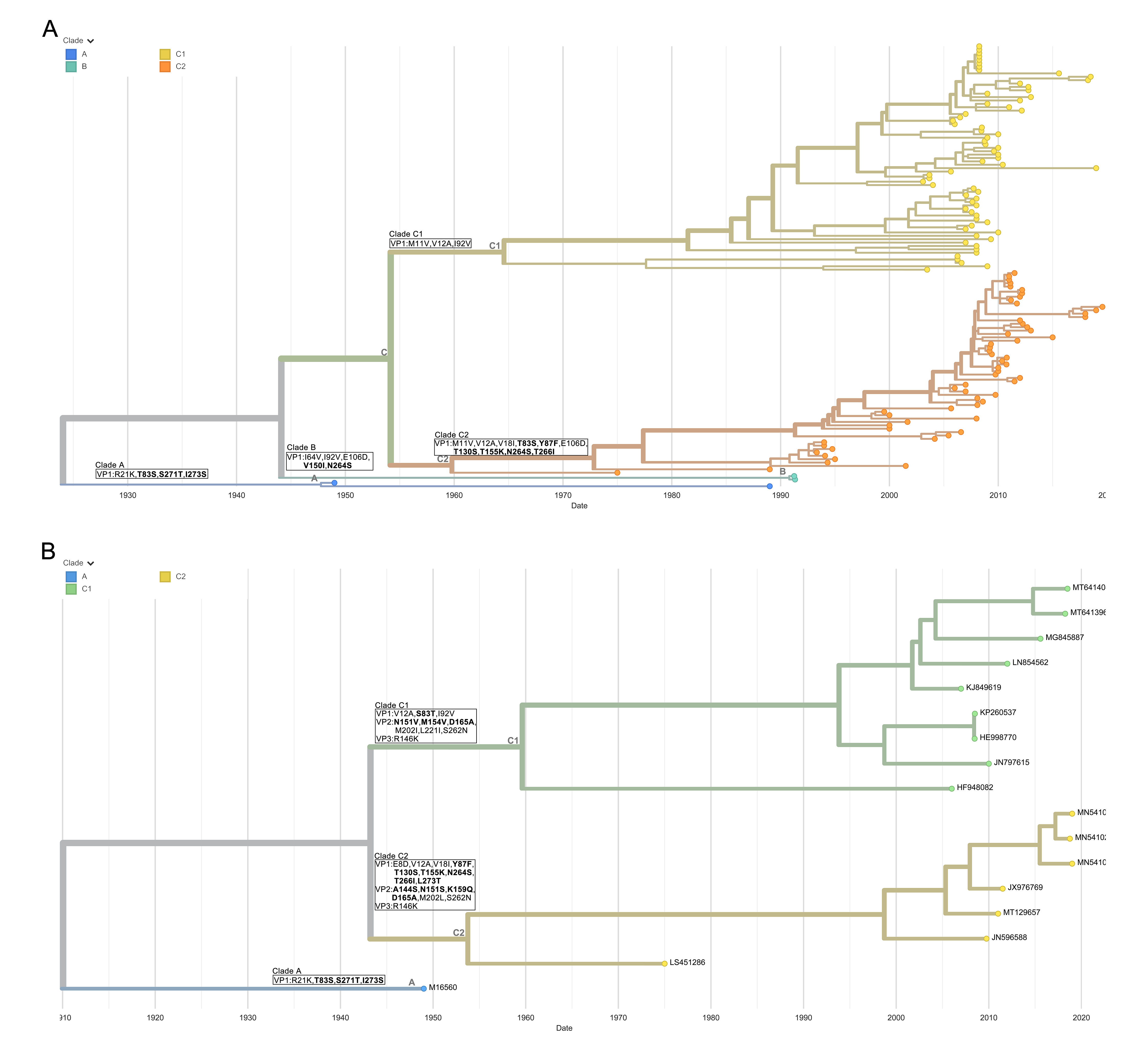

Supplement: S5 Fig — CVB1 time-scale phylogenetic trees based on VP1 sequences (A) and genome sequences (B). (TIF) [file pone.0290584.s005.tif]

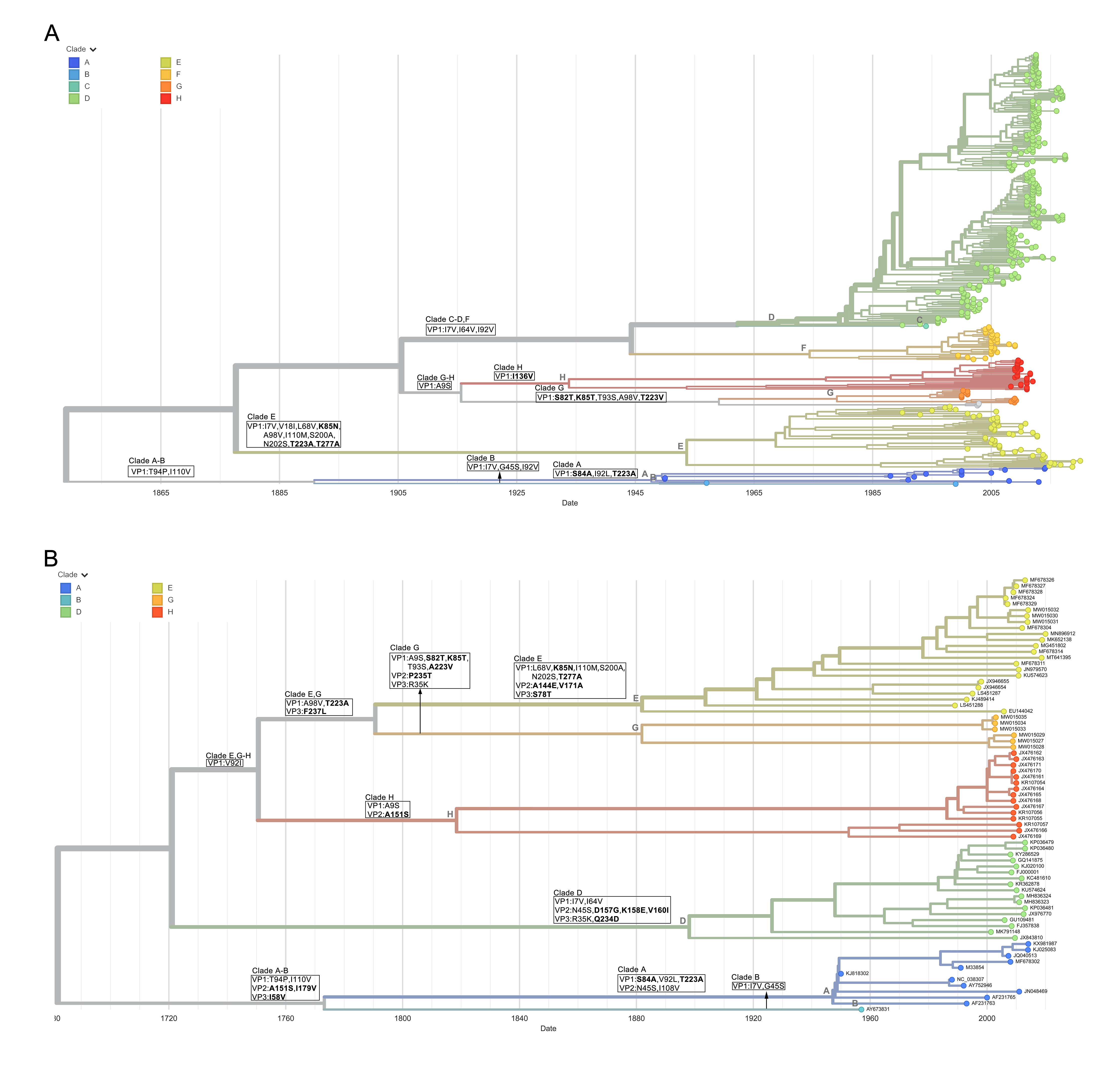

Supplement: S6 Fig — CVB3 time-scale phylogenetic trees based on VP1 sequences (A) and genome sequences (B). (TIF) [file pone.0290584.s006.tif]

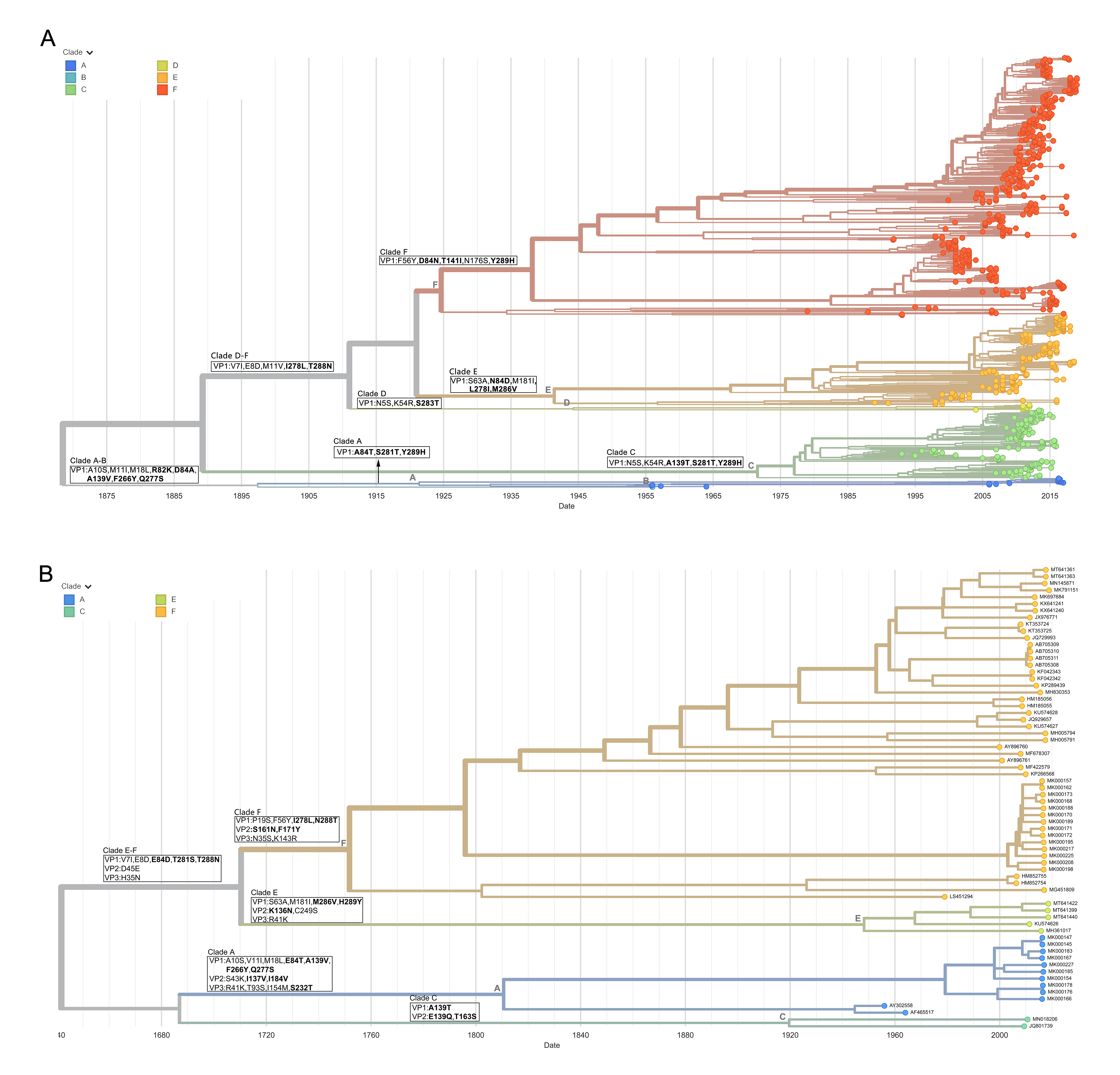

Supplement: S7 Fig — E6 time-scale phylogenetic trees based on VP1 sequences (A) and genome sequences (B). (TIF) [file pone.0290584.s007.tif]

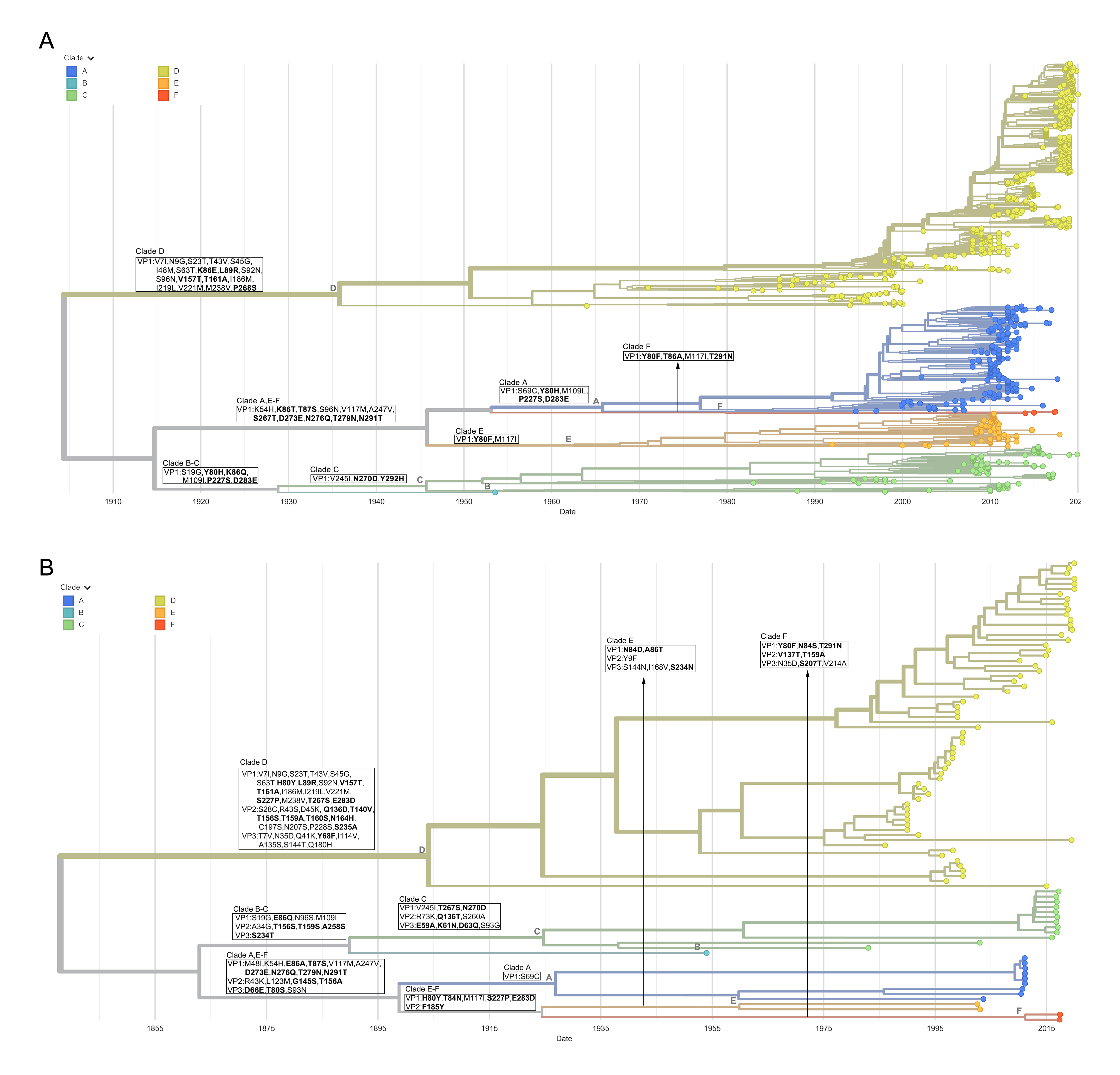

Supplement: S8 Fig — E11 time-scale phylogenetic trees based on VP1 sequences (A) and genome sequences (B). (TIF) [file pone.0290584.s008.tif]

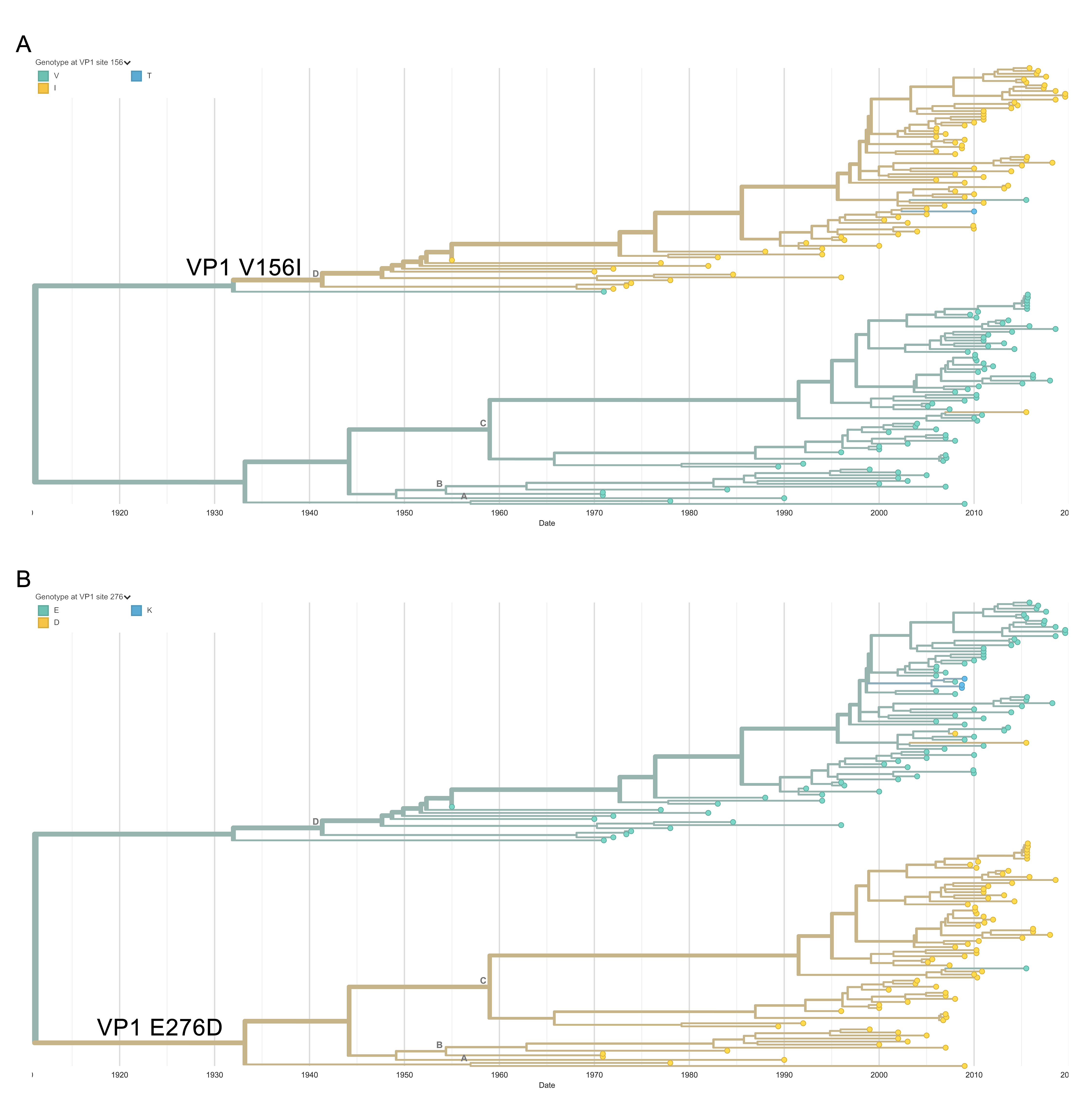

Supplement: S9 Fig — (TIF) [file pone.0290584.s009.tif]
