# Supplementary material for: In silico epitope prediction and evolutionary analysis reveals capsid mutation patterns for enterovirus B
Source: PLoS One. 2023 Aug 28;18(8):e0290584. doi: 10.1371/journal.pone.0290584 (PMC10461833; doi:10.1371/journal.pone.0290584)
Supplement: S1 Table — (DOCX) [file pone.0290584.s010.docx]

**S1 Table. Number of EVB sequences for evolutionary analysis.**

| EVB | VP1 | Genome |
| --- | --- | --- |
| E6 | 890 | 68 |
| E11 | 908 | 90 |
| E30 | 1763 | 114 |
| CVB1 | 131 | 17 |
| CVB3 | 428 | 73 |
| CVB5 | 795 | 153 |
