# Supplementary material for: In silico epitope prediction and evolutionary analysis reveals capsid mutation patterns for enterovirus B
Source: PLoS One. 2023 Aug 28;18(8):e0290584. doi: 10.1371/journal.pone.0290584 (PMC10461833; doi:10.1371/journal.pone.0290584)
Supplement: S2 Table — The upper triangle shows the sequence identity (%) of the viral proteins (VP1-VP3), and the lower triangle shows their 3D structural differences (RMSD, Å). (DOCX) [file pone.0290584.s011.docx]

**S2 Table. Sequence identities (%) and RMSDs (Å) of EVB viral proteins.**

| EVB | E6 | E11 | E30 | CVB1 | CVB3 | CVB5 |
| --- | --- | --- | --- | --- | --- | --- |
| E6 | - | 74.52 | 76.21 | 76.89 | 74.65 | 75.10 |
| E11 | 0.545 | - | 70.92 | 75.22 | 72.99 | 75.16 |
| E30 | 0.425 | 0.724 | - | 73.21 | 73.62 | 73.47 |
| CVB1 | 0.530 | 0.339 | 0.723 | - | 81.57 | 82.52 |
| CVB3 | 0.586 | 0.826 | 0.417 | 0.873 | - | 80.23 |
| CVB5 | 0.459 | 0.740 | 0.326 | 0.733 | 0.347 | - |

The upper triangle shows the sequence identity (%) of the viral proteins (VP1-VP3), and the lower triangle shows their 3D structural differences (RMSD, Å).
