# Supplementary material for: In silico epitope prediction and evolutionary analysis reveals capsid mutation patterns for enterovirus B
Source: PLoS One. 2023 Aug 28;18(8):e0290584. doi: 10.1371/journal.pone.0290584 (PMC10461833; doi:10.1371/journal.pone.0290584)
Supplement: S3 Table — *The same mutations indicate parallel mutations or reverse mutations in different clades. (DOCX) [file pone.0290584.s012.docx]

**S3 Table. Summary of clade mutations of EVB genome phylogenetic trees.**

| VPs | Secondary structure | E6 | E11 | E30 | CVB1 | CVB3 | CVB5 |
| --- | --- | --- | --- | --- | --- | --- | --- |
| VP1 | N-terminus | V7I,E8D,  A10S,V11I,  M18L,P19S,  F56Y,S63A | V7I,N9G,  S19G,S23T,  T43V,S45G,  M48I,K54H,  S63T,S69C | G5S,S5N,S5G,  K9N,K9R,  I18V,I18V,  V23T,Q27R,  V54I,V54I,  F56Y,L64I | E8D,V12A,  V12A,V18I,  R21K | I7V^*^,I7V^*^,A9S,  A9S,G45S,  I64V,L68V | V7I,I7V,G19S,  A45S |
|  | BC loop | E84T,E84D | H80Y,H80Y,  Y80F,N84D,  T84N,N84S,  E86Q,E86A,  A86T,T87S,  L89R | A84V,D87E | S83T,T83S,  Y87F | S82T,S84A,  K85T,K85N | N85D,F89I |
|  | DE loop | A139T,A139V |  |  | T130S |  |  |
|  | EF loop |  | V157T,T161A | K156R,G157S | T155K |  | V156I |
|  | HI loop |  | S227P,S227P |  |  | T223A,T223A,  A223V |  |
|  | C-terminus | F266Y,Q277S,  I278L,T281S,  M286V,T288N,  N288T,H289Y | T267S,T267S,  N270D,D273E,  N276Q,T279N,  E283D,E283D,  N291T,T291N | R263G,K263R,  D268N,V269I,  E274D,G277S,  G277D,G277S,  G277N,S284T,  T285N,N287S,  P288A,V289M,V289L,L290K,  L290S,S290A,  S290T | N264S,T266I,  S271T,L273T,  I273S | T277A | E272K,S273G,  G273S,E276D,  A279T |
|  | Others | M181I | S92N,N96S,  M109I,M117I,  V117M,I186M,  I219L,V221M,  M238V,V245I,  A247V | H78Q,I120V,  I120V,I145V,  S204N,N234S,  N234D,K247R | I92V | V92L,V92I,  T93S,T94P,  A98V,I110V,  I110M,S200A,  N202S | Y75F,F75Y,  A90G,S95N,  V99A,M180I,  K200R |
| VP2 | EF loop | K136N,I137V,  E139Q,S161N,  T163S,F171Y,  I184V | Q136D,Q136T,  V137T,T140V,  G145ST,156A,  T156S,T156S,  T159A,T159A,  T159S,T160S,  N164H,F185Y | S156T,G158E,  E158D,Y185F | A144S,N151S,  N151V,M154V,  K159Q,D165A,  D165A | A144E,A151S,  A151S,D157G,  K158E,V160I,  V171A,I179V | N153S,T154M,  E156D,T160S,  F185Y |
|  | HI loop |  | S235A |  |  | P235T |  |
|  | Others | S43K,D45E,  C249S | Y9F,S28C,  A34G,R43S,  R43K,D45K,  R73K,L123M,  C197S,N207S,  P228S,A258S,  S260A | Y9F,V37E,  H45N,H45N,  H45R,S74T,  T104S,A104T | M202I,M202L,  L221I,S262N,  S262N | N45S,N45S,  I108V | T37V,E45D,  D45E,A104S,  V108I,I108V,  I223V,V223I |
| VP3 | N-terminus |  | E59A,K61N,  D63Q,D66E,  Y68F | E60G,H61N,  I65M,I65M,  I65M |  | I58V | T58I,T58A,  L63S,L63M,  L63M,A67S |
|  | BC loop |  | T80S | N77S |  | S78T |  |
|  | HI loop |  | S207T |  |  |  |  |
|  | C-terminus | S232T | S234T,S234N |  |  | Q234D,F237L | K232Q,D234E,  S235N,F237Y |
|  | Others | H35N,N35S,  R41K,R41K,  T93S,K143R,  I154M | T7V,N35D,  N35D,Q41K,  S93N,S93G,  I114V,A135S,  S144T,S144N,  I168V,Q180H,  V214A | Q35H,I46V,  Y91H,97R,  H97R,K146R | R146K,R146K | R35K,R35K | E35A,E35D,  D35N,T88I,  I103V,M181V,  M181T,I190V |

^*^The same mutations indicate parallel mutations or reverse mutations in different clades.
