# Supplementary material for: In silico epitope prediction and evolutionary analysis reveals capsid mutation patterns for enterovirus B
Source: PLoS One. 2023 Aug 28;18(8):e0290584. doi: 10.1371/journal.pone.0290584 (PMC10461833; doi:10.1371/journal.pone.0290584)
Supplement: S4 Table — (DOCX) [file pone.0290584.s013.docx]

**S4 Table. Proportion of epitope and VP1 N-terminus mutations in clade mutations (%).**

| Regions | E6 | E11 | E30 | CVB1 | CVB3 | CVB5 |
| --- | --- | --- | --- | --- | --- | --- |
| VP1 N-terminus+  epitope | 71.79 | 61.46 | 67.65 | 73.33 | 66.67 | 54.00 |
| epitope | 51.28 | 51.04 | 48.53 | 56.67 | 50.00 | 46.00 |
| VP1 N-terminus+  VP1 C-terminus+  VP2 EF loop | 58.97 | 35.42 | 52.94 | 56.67 | 38.10 | 28.00 |
