# Supplementary material for: In silico epitope prediction and evolutionary analysis reveals capsid mutation patterns for enterovirus B
Source: PLoS One. 2023 Aug 28;18(8):e0290584. doi: 10.1371/journal.pone.0290584 (PMC10461833; doi:10.1371/journal.pone.0290584)
Supplement: Attachment — Submitted filename: reviewer comments - Mai-17062023 - Copy.docx [file pone.0290584.s014.docx]

**Reviewer Comments**

Dear Editor(s),	June 17, 2023

I am pleased to submit my comments for the submitted manuscript (**PONE-D-23-14925)** entitled “**In silico epitope prediction and evolutionary analysis reveals capsid mutation patterns for enterovirus B”** to be taken into consideration for publication by your esteemed journal “***PLOS ONE***”.

Enterovirus B (EVB) is common species of enterovirus, mainly consisting of Echovirus (Echo) and Coxsackievirus B (CVB). The population, especially among children, is generally susceptible to EVB. Since the 21st century, EVB has been widely prevalent in the world, which can cause serious diseases, such as viral meningitis, myocarditis, and neonatal sepsis. In recent years, severe EVB related outbreaks had occurred in many countries around the world, suggesting the need to strengthen monitoring and early warning of EVB. By using cryo-electron microscopy, the three-dimensional structure of EVBs and their uncoating receptors (FcRn and CAR) have been determined, laying the foundation for the study of viral pathogenesis and therapeutic antibodies. A limited number of epitopes bound to neutralizing antibodies have also been determined, but it is not clear whether more epitopes are present or whether epitope variation plays a key role in molecular evolutionary history and epidemics, as in influenza and SARS-CoV-2. Therefore, this study aimed to systematically predict the epitopes of EVB based on the 3D structures and to analyse the role of epitope mutations in the molecular evolution and epidemic, so as to provide references for the monitoring and early warning of EVB outbreaks and the development of therapeutic antibodies

In this study, the authors have successfully investigated the conformational epitopes of six representative EVB serotypes (E6, E11, E30, CVB1, CVB3 and CVB5) by using the bioinformatics-based epitope prediction algorithm. The epitopes of EVB were found to be distributed into three clusters, where the VP1 BC loop, C-terminus and VP2 EF loop were the main regions of EVB epitopes. Among them, the VP1 BC loop and VP2 EF loop may be the key epitope regions that determined the use of the uncoating receptors. Further molecular evolution analysis based on the VP1 and genome sequences showed that the VP1 C-terminus and VP2 EF loop as well as a potential “breathing epitope” VP1 N-terminus were common mutation hotspot regions, suggesting that the emergence of evolutionary clades was drived by epitope mutations. Finally, footprints showed mutations were located on or near epitopes, while mutations on the receptor binding sites were rare. The bioinformatics study of EVB epitopes will provide important information for the monitoring and early warning of EVB epidemics and the development of therapeutic antibodies.

The authors have done good work on the title ““**In silico epitope prediction and evolutionary analysis reveals capsid mutation patterns for enterovirus B**”. It will add new knowledge and new areas of research to the subject area compared with other published material.

**However, i have some minor concerns:**

1. **Moderate editing is required throughout the manuscript, for example:**
1. In the introduction section, “According to the 2014-2016 surveillance report of the US Centers for Disease Control and Prevention, six of the top ten enteroviruses most frequently causing outbreaks belonged to EVB”. Moderate editing is required.
2. In the introduction section, “The uncoating receptors of Echo and CVB are Human Neonatal Fc Receptor (FcRn) and Coxsackie-adenovirus receptor (CAR), respectively both of which bind to the virus by inserting into the canyon.”. Moderate editing is required.
3. In the introduction section “The nAbs 4B10 and 6C5 bind to the interior and northern rim of the canyon of E30 respectively, both of which block the specific binding of E30 to FcRn and thus exert neutralizing activity.” Moderate editing is required.
4. In the section of Conformational epitopes prediction, “Now we use it to predict the epitopes of EVB.”. Moderate rephrasing is required.
5. In the section of Conformational epitopes prediction, “Constructing the multiple chains. Each chain consisted of VP1-VP3 and Chain 2/3/4 surrounded by the central chain Chain 1. (2) Predicting epitopes of EVB based on the multiple chains by the three algorithms (Epitopia, Ellipro, and DiscoTope) [26-28]. (3) Screening the amino acid residues on the relatively exposed surface of the viral capsid from Chain 1. Those residues, simultaneously predicted by all three algorithms, were defined as core epitopes. The surrounding epitopes were required to meet two conditions: (1) Be Predicted as candidate epitopes by any two algorithms; (2) B”. Moderate editing is required.

Best regards,

Dr. Mai Abdel Haleem Abusalah

Faculty of Medical Allied Science,

Zarqa University,

Zarqa, 13110, Jordan.

Tel: +962-796862347

e-mail: [ellamomo88@yahoo.com](mailto:ellamomo88@yahoo.com)
